# Evaluation of preoperative difficult airway prediction methods for adult patients without obvious airway abnormalities: a systematic review and meta-analysis

**DOI:** 10.1186/s12871-024-02627-1

**Published:** 2024-07-17

**Authors:** Zhichen Wang, Yile Jin, Yueying Zheng, Hanjian Chen, Jingyi Feng, Jing Sun

**Affiliations:** 1 Department of Clinical Engineering and Material Supplies, The First Affiliated Hospital, Zhejiang University School of Medicine, No.79 Qingchun Road, Hangzhou, 310003 China; 2 Department of Anesthesiology, The First Affiliated Hospital, Zhejiang University School of Medicine, Hangzhou, Zhejiang, 31003 China

**Keywords:** Airway management, Difficult intubation, Prediction, Ultrasound, Difficult airway

## Abstract

**Background:**

This systematic review aims to assist clinical decision-making in selecting appropriate preoperative prediction methods for difficult tracheal intubation by identifying and synthesizing literature on these methods in adult patients undergoing all types of surgery.

**Methods:**

A systematic review and meta-analysis were conducted following PRISMA guidelines. Comprehensive electronic searches across multiple databases were completed on March 28, 2023. Two researchers independently screened, selected studies, and extracted data. A total of 227 articles representing 526 studies were included and evaluated for bias using the QUADAS-2 tool. Meta-Disc software computed pooled sensitivity (SEN), specificity (SPC), positive likelihood ratio (PLR), negative likelihood ratio (NLR), and diagnostic odds ratio (DOR). Heterogeneity was assessed using the Spearman correlation coefficient, Cochran’s-Q, and I^2^ index, with meta-regression exploring sources of heterogeneity. Publication bias was evaluated using Deeks’ funnel plot.

**Results:**

Out of 2906 articles retrieved, 227 met the inclusion criteria, encompassing a total of 686,089 patients. The review examined 11 methods for predicting difficult tracheal intubation, categorized into physical examination, multivariate scoring system, and imaging test. The modified Mallampati test (MMT) showed a SEN of 0.39 and SPC of 0.86, while the thyromental distance (TMD) had a SEN of 0.38 and SPC of 0.83. The upper lip bite test (ULBT) presented a SEN of 0.52 and SPC of 0.84. Multivariate scoring systems like LEMON and Wilson’s risk score demonstrated moderate sensitivity and specificity. Imaging tests, particularly ultrasound-based methods such as the distance from the skin to the epiglottis (US-DSE), exhibited higher sensitivity (0.80) and specificity (0.77). Significant heterogeneity was identified across studies, influenced by factors such as sample size and study design.

**Conclusion:**

No single preoperative prediction method shows clear superiority for predicting difficult tracheal intubation. The evidence supports a combined approach using multiple methods tailored to specific patient demographics and clinical contexts. Future research should focus on integrating advanced technologies like artificial intelligence and deep learning to improve predictive models. Standardizing testing procedures and establishing clear cut-off values are essential for enhancing prediction reliability and accuracy. Implementing a multi-modal predictive approach may reduce unanticipated difficult intubations, improving patient safety and outcomes.

**Supplementary Information:**

The online version contains supplementary material available at 10.1186/s12871-024-02627-1.

## Background

### Rational

With the rapid development of anesthesia-related technologies, breakthrough devices such as video laryngoscopes and supraglottic airway devices(SGAs) have greatly facilitated the work of anesthesiologists and other healthcare workers in airway management [1]. However, difficult airway management remains a major challenge for anesthesiologists. Difficult airways refer to clinical situations where skilled healthcare professionals encounter difficulties when using tools such as face masks or tracheal intubation stylets for ventilation [2]. The occurrence of difficult airways means that unconscious patients may suffer irreversible brain damage or even death due to inadequate oxygen supply or ventilation. Moreover, research has found that more than 30% of serious anesthesia-related complications are caused by improper airway management [3]. Therefore, accurately predicting the possibility of difficult airway occurrence before surgery can ensure that anesthesiologists make sufficient preoperative preparations and anticipate the occurrence of difficult airways, so as to respond promptly when it occurs.

Currently, various types of methods have been proposed for predicting difficult airways. This article mainly analyzes the prediction methods for difficult tracheal intubation. There are three main categories: physical examination, multivariate scoring system and imaging test. However, multiple studies have shown significant differences in the accuracy and reliability of these methods. For example, one study showed that using the modified Mallampati score to predict difficult airway intubation had a sensitivity (SEN) of 0.96 and specificity (SPC) of 0.55 [4]. However, another showed completely opposite results with a SEN of 0.38 and SPC of 0.9 [5]. Therefore, conducting a meta-analysis to assess the effectiveness of various prediction methods and providing decision-making references for clinical practice has become particularly important.

### Objective

This systematic review aims to assist clinical decision-making in selecting appropriate preoperative prediction methods for difficult tracheal intubation by identifying and synthesizing literature on these methods in adult patients undergoing all types of surgery.

## Methods

### Registration

We conducted a systematic review and meta-analysis on diagnostic test accuracy following the PRISMA guidelines [6]. Before screening literature, we developed and registered a review protocol in PROSPERO (registration number: CRD42023412075; accessed March 28th, 2023) to guide the entire process.

### Eligibility Criteria

This meta-analysis of diagnostic accuracy will only include studies that meet specific criteria. Eligible studies must have aimed to evaluate the accuracy of one or more methods for predicting difficult tracheal intubation and provided accuracy data, such as true positive [7]. Additionally, studies must have been published in Chinese or English and included a study population of adults aged 16 years or older with no apparent airway abnormalities who underwent general tracheal intubation using a standard laryngoscope [8]. Studies with incomplete data or populations with airway abnormalities, rapid sequence intubation during surgery, or history of difficult airways will be excluded. Comments, editorials, conference abstracts, reviews, meta-analyses, or case reports will also not be included.

Given that there is no universally accepted definition for difficult tracheal intubation, this meta-analysis adopts the definitions used by the researchers in each included study. Specifically, difficult tracheal intubation is defined either by a Cormack-Lehane grade III or IV classification, which indicates difficulty in visualizing the vocal cords during laryngoscopy, or by the need for several attempts to successfully intubate. This approach ensures inclusivity of various operational definitions used in the current literature and allows for a comprehensive analysis of the predictive methods [8].

### Information Sources and Search Methods

This meta-analysis conducted a comprehensive electronic search on March 28, 2023, from the following databases: China National Knowledge Infrastructure (CNKI), Wanfang Database, Embase, PubMed, and Cochrane Library. The literature lists of eligible studies and relevant review articles were also screened. There was no publication date limit for this selection.

The search strategy used was as follows: ((((((((((test[Title/Abstract]) OR (tests[Title/Abstract])) OR (exam[Title/Abstract])) OR (examination[Title/Abstract])) OR (predict[Title/Abstract])) OR (predictor[Title/Abstract])) OR (assessment[Title/Abstract])) OR (exam[Title/Abstract])) OR (physical examination [ Title / Abstract])) or management [ Title / Abstract]) AND ((((((difficult airway [ Title / Abstract]) or difficult intubation [ Title / Abstract]) or difficult face mask ventilation [ Title / Abstract]) or difficult laryngoscopy [ Title / Abstract]) Or difficult tracheal intubation [ title / abstract])) or airway management [ title / abstract]).

### Study Selection

Two researchers (ZW and YJ) conducted independent screenings. The first round assessed the relevance of abstracts and titles, while the second round confirmed selected studies' relevance and compliance with inclusion criteria. Any uncertainties or disagreements were resolved through consensus or judgment from a third researcher (JS).

### Data Collection Process

Two researchers (ZW and YJ) independently extracted and calculated data from each included studies in standardized tables in Microsoft Excel. Any uncertainties or disagreements during the data collection process were resolved through consensus or judgment from a third independent researcher (JS).

### Data Items

During the data collection process, two researchers (ZW and YJ) independently collect the following data from each included study: author name, publication year, research location, research design and methods, patient demographic, sample size, difficult tracheal intubation prevalence rate, ultrasound measurement indicators, cut-off values of ultrasound measurement indicators, accuracy data, sensitivity, and specificity. If the research involves multiple prediction methods or multiple data for a single method, each set of data will be recorded as an individual study.

### Risk of *Bias* in Individual Studies

Two researchers (ZW and YL) used the revised version of the Quality Assessment of Diagnostic Accuracy Studies 2 (QUADAS-2) tool to independently assess the quality of all included studies. This assessment process was conducted using Review Manager 5. Any uncertainties or disagreements during this process were resolved through consensus or judgment from a third independent researcher (JS).

## Summary Measures and Planned Methods of Analysis

We used Meta-Disc statistical software version 1.4 to analyze the data [9]. For the meta-analysis, we computed SEN, SPC, PLR, NLR, and DOR for each eligible study using accuracy data.

We assessed heterogeneity by calculating the Spearman correlation coefficient and examining the summary receiver operating characteristic (SROC) curve for a "shoulder-arm" point distribution [10]. A strong positive correlation or a "shoulder-arm" point distribution indicates a threshold effect. We also used Cochran's-Q value and I [2] index to identify non-threshold heterogeneity, with p-values ≤ 0.1 indicating significant heterogeneity. If there was no heterogeneity among studies, we used a fixed-effect model for meta-analysis; otherwise, we used a random-effects model instead. We calculated pooled sensitivity, specificity, positive likelihood ratio (PLR), negative likelihood ratio (NLR), and diagnostic odds ratio (DOR) along with their respective 95% confidence intervals based on whether there was heterogeneity or not. Additionally, we plotted an SROC curve to determine its area under the curve (AUC) and Q* index [11]. We employed meta-regression analysis to further examine potential sources of heterogeneity.

We used Deeks' funnel plot method in STATA version 17.0 with the MIDAS module to assess publication bias [12]. A p-value below 0.05 suggests the presence of significant publication bias.

## Results

### Study Selection

**Fig. 1:**
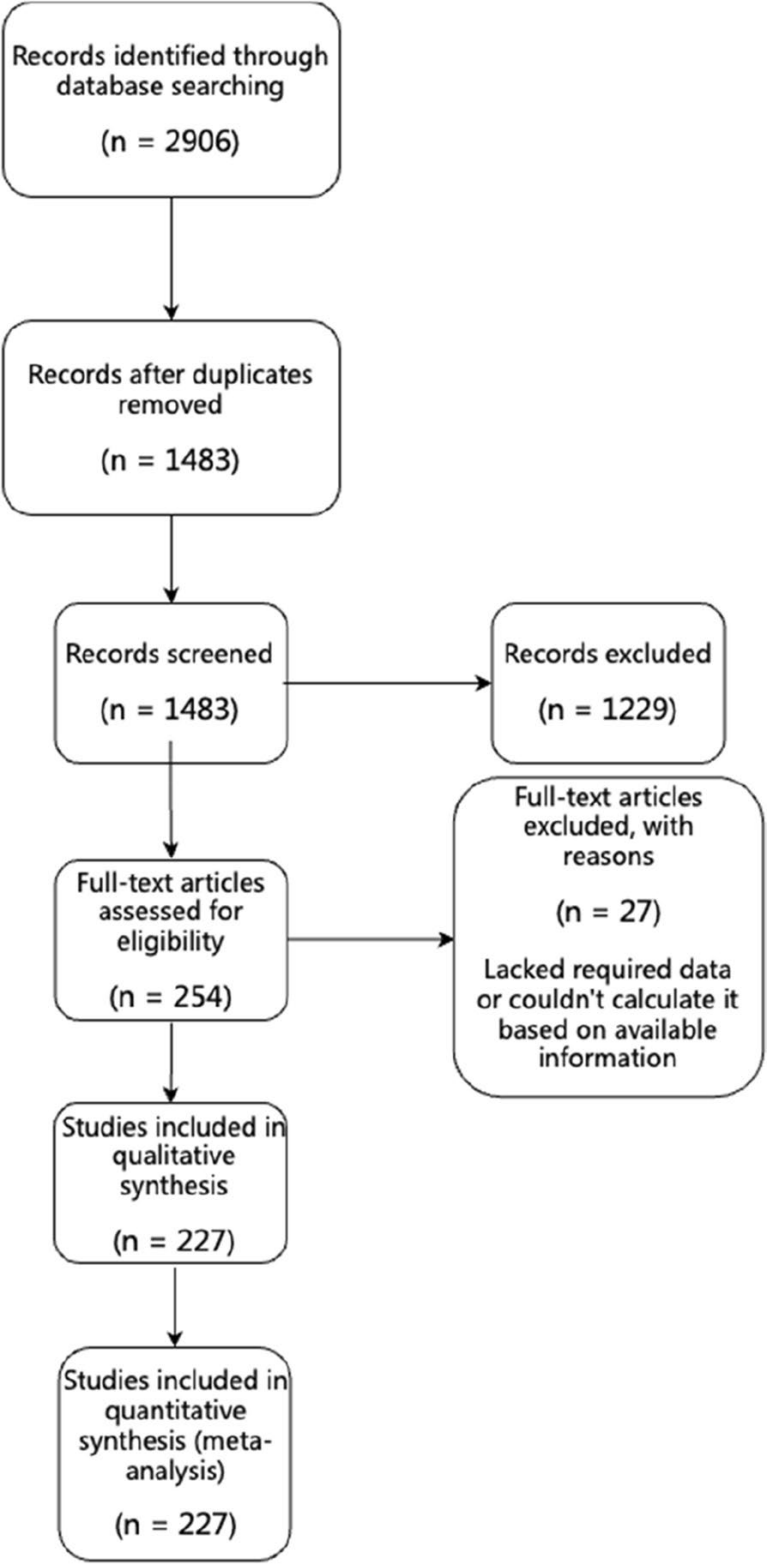
Flow diagram of included and excluded studies

We retrieved 2906 articles through a literature search in multiple databases. After excluding 1423 duplicates, we were left with 1483 articles. We screened the titles and abstracts of these articles, excluding 1229 for reasons such as unrelated content (1198 studies), literature reviews/meta-analyses/comments/editorials (26 articles), children as participants (3 articles), or mannequin/simulator studies (2 articles). This left us with 254 remaining articles. In the second round of screening, we evaluated full-text papers and excluded another 27 that lacked required data or couldn't calculate it based on available information. Ultimately, our meta-analysis included 227 eligible studies involving a total of 686,089 patients [4, 5, 13–234]. Fig. 1 summarizes our process for identifying, screening, and selecting literature.

### Study characteristics

In this study, 227 papers were analyzed, including 526 studies with a total of 686,089 patients. Of these patients, 37,836 had difficult tracheal intubation (prevalence rate of 5.51%). Most of the papers were published in English and the remaining 35 were published in Chinese [4, 47, 62, 67–69, 76, 80, 86, 87, 118, 120, 137, 138, 148, 153, 156, 164, 167, 168, 170, 173, 177, 178, 191, 193, 205–207, 212–214, 216, 221, 224]. Supplementary Table S1 summarizes the important characteristics of all included studies.

Most of these studies (159 articles) were conducted in Asia [4, 17, 19, 24, 26, 28, 30, 31, 33, 37, 39–42, 44, 47, 50, 51, 53–56, 62–64, 66–69, 71–73, 76–80, 83, 86, 87, 89, 90, 92, 95, 98–103, 105, 107–109, 112–123, 125, 127–134, 136–141, 143, 146–148, 150–153, 156, 158, 160–180, 182, 183, 185, 186, 188–193, 195, 196, 198–200, 203–217, 219, 221, 223–231, 233–235], mainly from India and China followed by Europe (38 articles) [5, 13, 15, 16, 18, 23, 27, 32, 34, 38, 43, 46, 49, 58, 65, 74, 75, 81, 82, 84, 85, 88, 91, 93, 97, 111, 126, 135, 149, 154, 157, 159, 181, 187, 202, 218], North America (22 articles) [14, 20–22, 25, 29, 35, 36, 57, 59–61, 70, 94, 96, 106, 124, 142, 144, 145, 155, 194], Africa (7 articles) [45, 48, 52, 184, 197, 201, 222] and South America (1 article) [232]. One hundred seventy-eight papers used prospective design, twelve used retrospective design, eighteen papers used case–control design. Sixty-nine papers used blinded experiment [15, 20, 25, 29–31, 44, 50, 57, 60, 61, 63, 71, 75, 77, 86, 90, 91, 95, 98, 102, 112, 115, 116, 123–125, 127–129, 139, 140, 143, 147, 152, 159–161, 169, 171–174, 179–183, 185, 186, 188–192, 196, 201, 204, 208, 209, 211, 218, 228–230, 234]. Twenty-four specifically selected obese populations for research [36, 39, 40, 43, 59, 61, 65, 70, 86, 99, 106, 109, 121, 131, 135, 142, 151, 154, 186, 187, 192, 209, 226] while some excluded obese populations.

Over 50% of the tests were conducted on the day of surgery in the operating room, while 18 were tested one to two days before. While most studies reported sensitivity and specificity for each prediction method, some only recorded accuracy data.

Regarding the prediction methods for difficult intubation, 210 studies used the modified Mallampati test, 128 studies used thyromental distance, 77 studies used upper lip bite test, 25 studies used Wilson's risk score, 9 studies used LEMON, 8 studies used El-Ganzouri risk index, 17 studies utilized ultrasound to measure the distance from the skin to the epiglottis, 10 studies measured the distance from skin to hyoid bone using ultrasound, 9 studies measured the distance from skin to vocal cords using ultrasound, and 7 studies used ultrasound to measure the hyomental distance ratio. Furthermore, 5 studies utilized ultrasound measurements for the ratio between the depth of pre-epiglottic space and the distance from epiglottis to vocal cord.

### Risk of *Bias* Within Studies

**Fig. 2:**
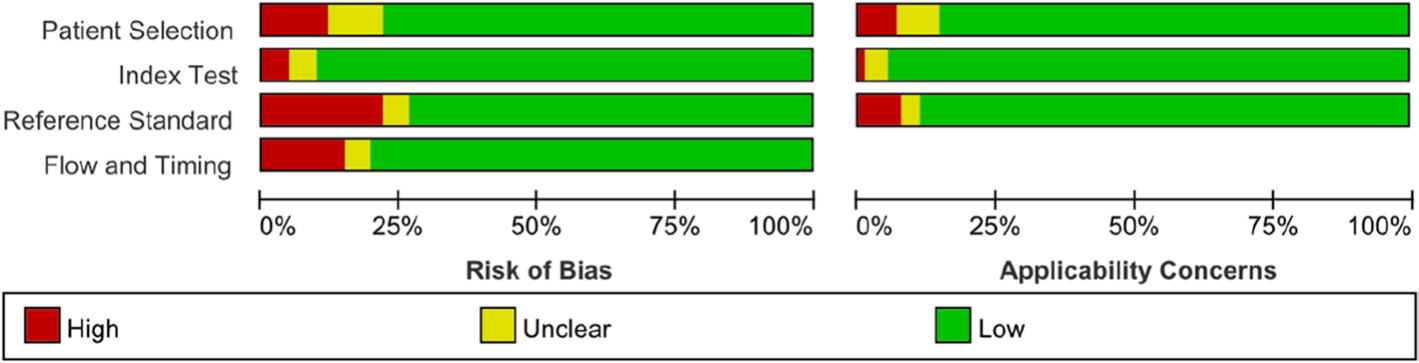
graphical summary of the risk of bias and applicability

The studies' quality was assessed using QUADAS-2, and the findings are presented in Fig. 2. Almost all studies indicated that difficult tracheal intubation assessment was performed before surgery. Only 69 articles explicitly used blinded methods [15, 20, 25, 29–31, 44, 50, 57, 60, 61, 63, 71, 75, 77, 86, 90, 91, 95, 98, 102, 112, 115, 116, 123–125, 127–129, 139, 140, 143, 147, 152, 159–161, 169, 171–174, 179–183, 185, 186, 188–192, 196, 201, 204, 208, 209, 211, 218, 228–230, 234]. When assessing the risk of bias in 227 studies using the QUADAS-2 tool, 27 studies showed problems with patient selection, 10 studies showed problems with index testing, 49 studies showed problems with reference standards, and 33 studies showed problems with procedures and timing. High risk factors were mainly due to unclear patient screening criteria or lack of blinded experiments in some studies.

### Results of Studies by prediction methods

This study examined 11 methods for predicting difficult tracheal intubation, which were selected through literature screening and can be categorized into three types: physical examination, multivariate scoring system, and imaging test. The methods include thyromental distance(TMD), upper lip bite test(ULBT), modified Mallampati test(MMT)LEMON, Wilson’s risk socre(WRS), El-Ganzouri risk index(EGRI), distance from the skin to the epiglottis measured using ultrasound (US-DSE), distance from skin to the hyoid bone measured using ultrasound(US-DSHB), distance from skin to the vocal cords measured using ultrasound(US-DSVC), hyomental distance ratio measured by ultrasound(US-HMDR) and the ratio of the depth of the pre-epiglottic space to the distance between the epiglottis and vocal cords measured using ultrasound (US- Pre-E/E-VC). Table 1 provides detailed information on each method.

**Table 1:**
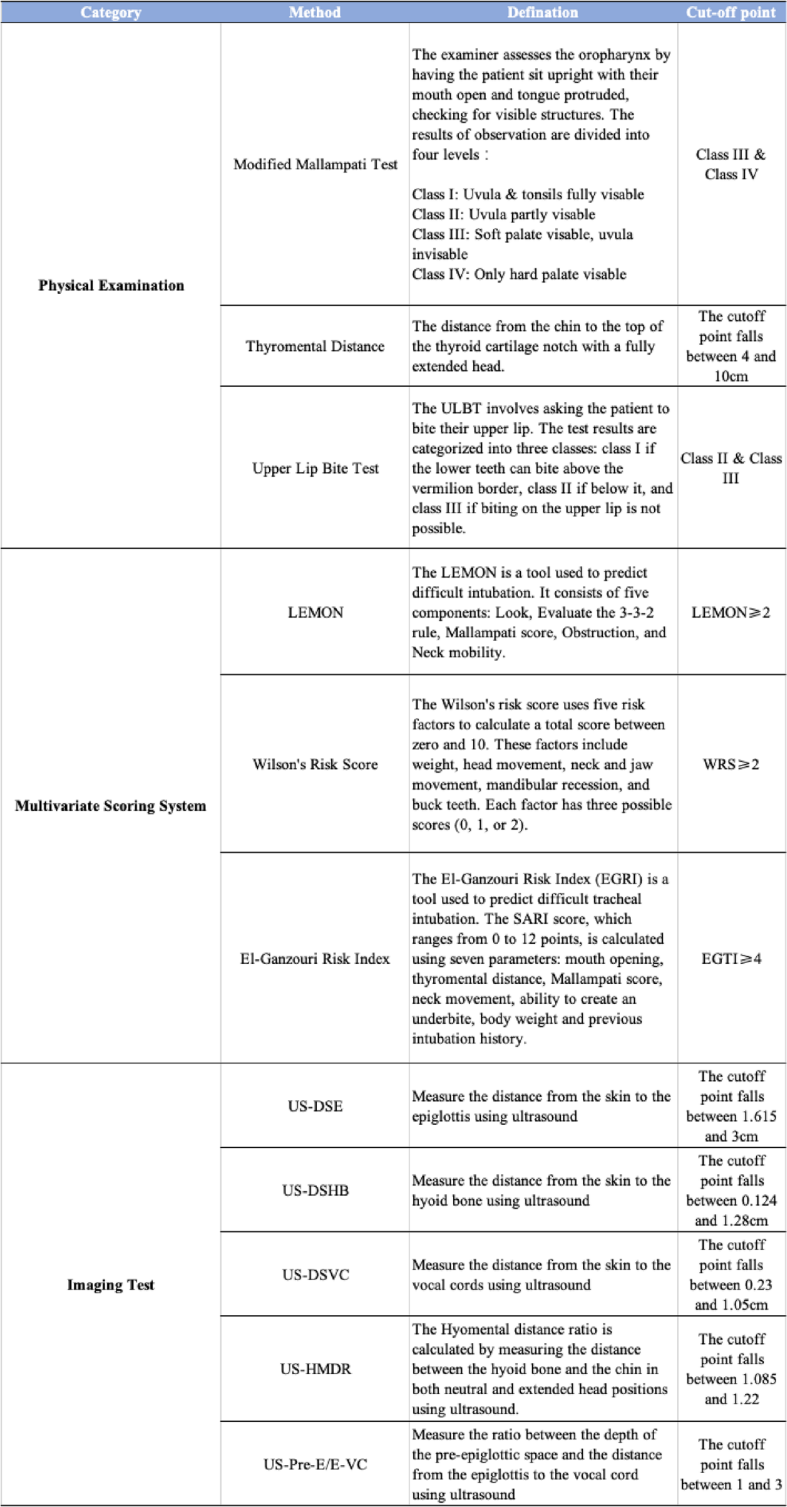
Detailed information on included prediction methods

#### Physical examination

**Fig. 3:**
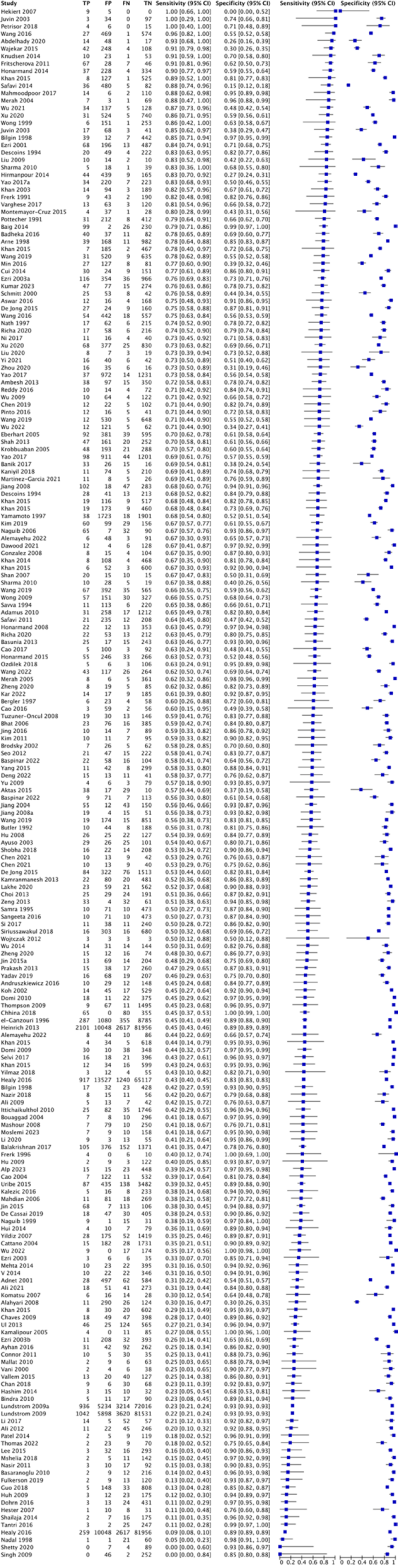
Forest plot of modified Mallampati test

For modified Mallampati test, this study analyzed 210 studies involving 532,526 patients, of which there were 25,045 cases of difficult airway intubation. The pooled diagnostic characteristics of modified Mallampati test were as follow: SEN 0.39 (0.39–0.4), SPC 0.86 (0.86–0.86), PLR 2.29 (2.7–3.15), NLR 0.62 (0.6–0.65), DOR 5.59(5.05–6.19) and AUC of 0.7445, with a Q* index of 0.6889. See Fig. 3.

**Fig. 4:**
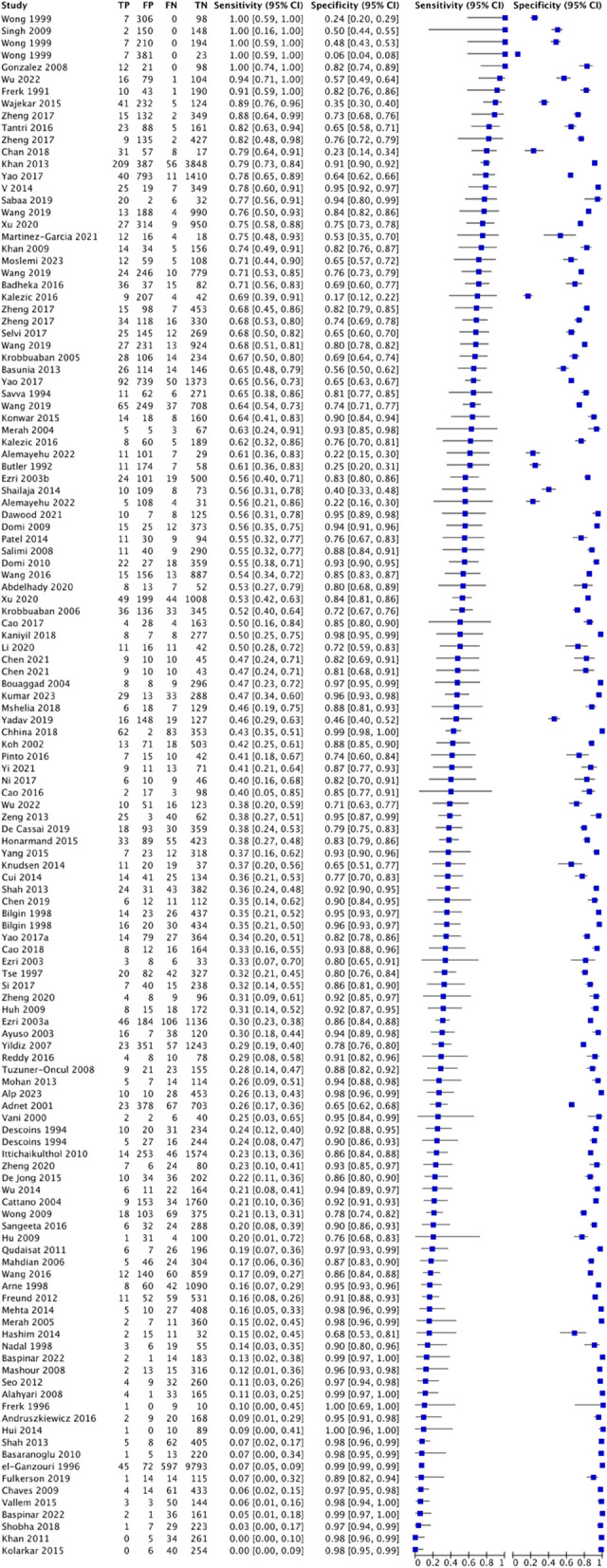
Forest plot of thyromental distance

For thyromental distance, this study analyzed 128 studies involving 68,603 patients, of which there were 5230 cases of difficult tracheal intubation. The pooled results showed a SEN of 0.38 (0.37-0.4), SPC of 0.83 (0.84-0.83), PLR of 2.78 (2.44-3.17), NLR of 0.72 (0.68-0.77), DOR of 4.51(3.69-5.51) and AUC of 0.7197, with a Q* index of 0.6687. See Fig 4.

**Fig. 5:**
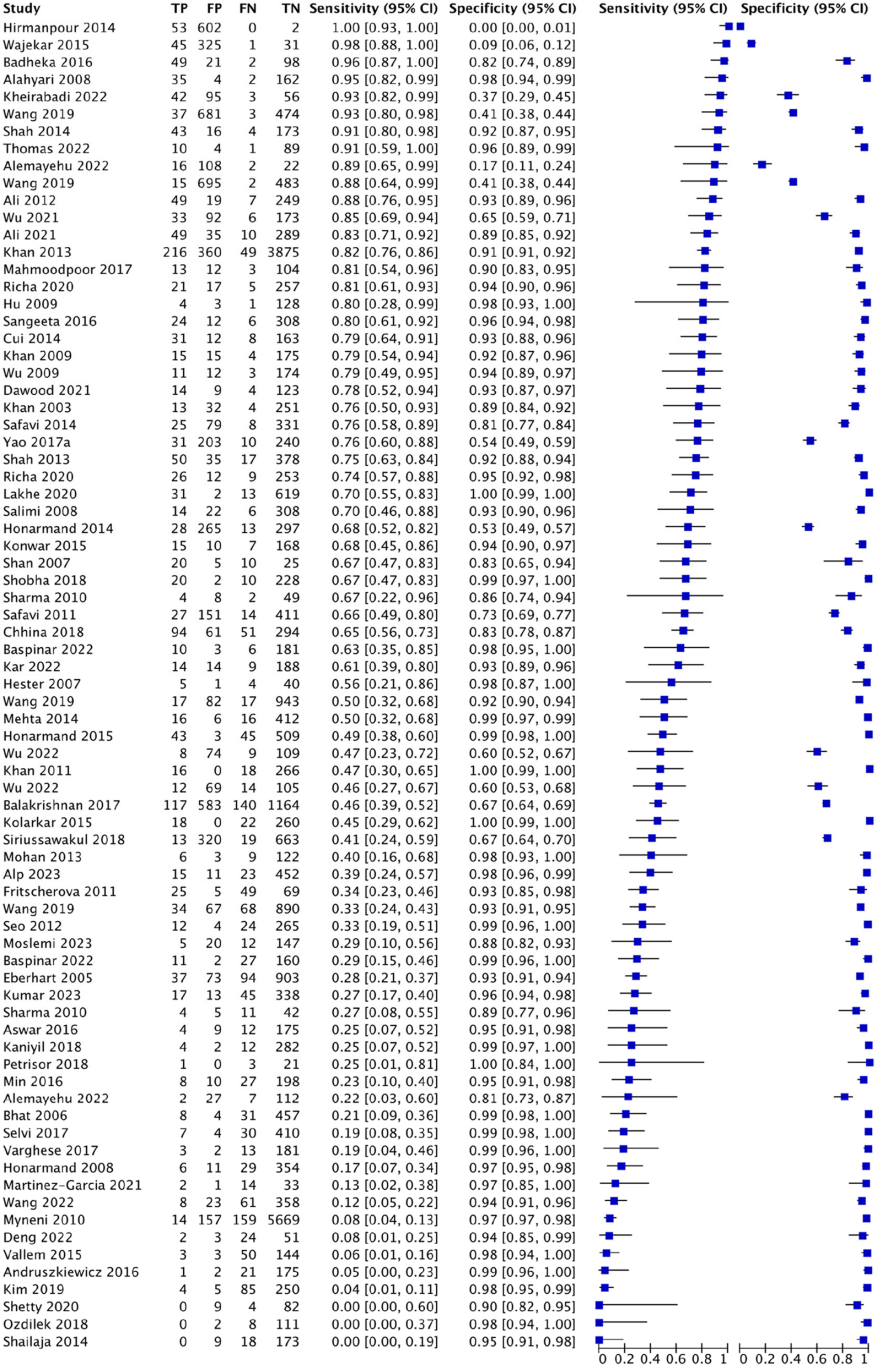
Forest plot of upper lip bite test

For upper lip bite test, this study analyzed 77 studies involving 38,164 patients, of which there were 3344 cases of difficult tracheal intubation. The pooled results showed a SEN of 0.52 (0.51–0.54), SPC of 0.84 (0.83–0.84), PLR of 6.54 (4.6–9.29), NLR of 0.51 (0.45–0.59), DOR of 15.15(10.6–21.65) and AUC of 0.8518, with a Q* index of 0.7829. See Fig. 5.

#### Multivariate scoring system

**Fig. 6:**
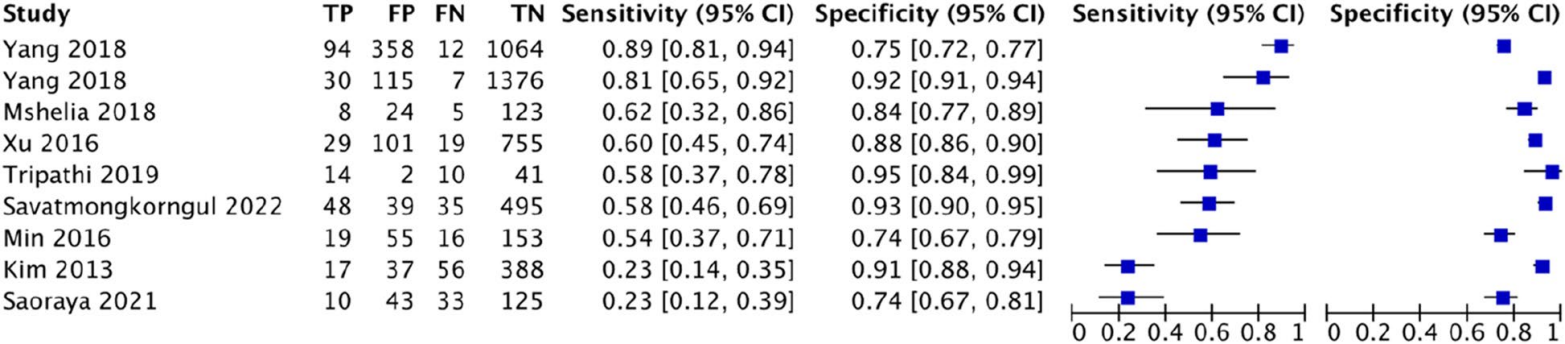
Forest plot of LEMON

For LEMON, this study analyzed 9 studies involving 5756 patients, of which there were 462 cases of difficult tracheal intubation. The pooled results showed a SEN of 0.58 (0.54–0.63), SPC of 0.85 (0.84–0.86), PLR of 3.99 (2.57–6.19), NLR of 0.46 (0.29–0.72), DOR of 9.01(3.99–20.32) and AUC of 0.8698, with a Q* index of 0.8003. See Fig. 6.

**Fig. 7:**
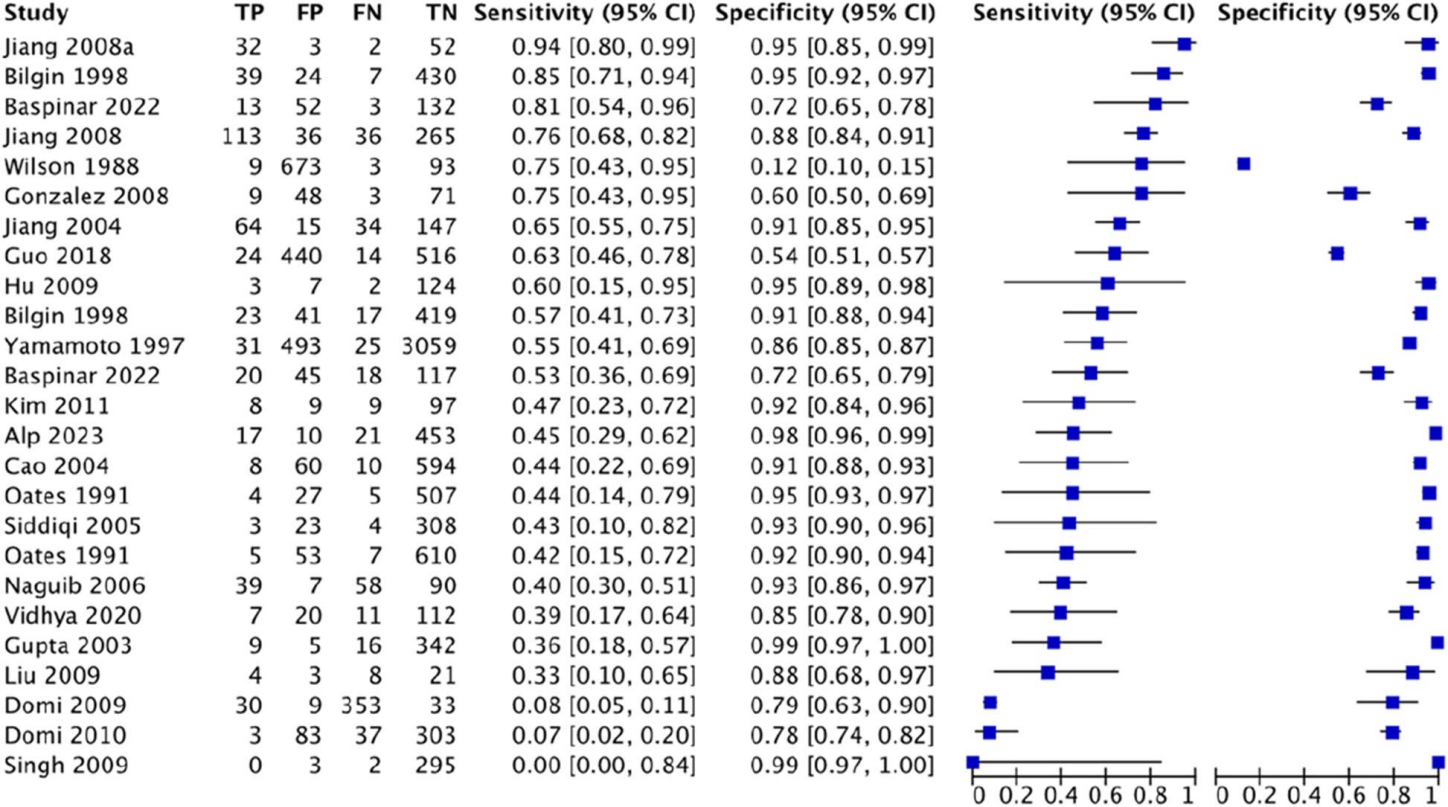
Forest plot of Wilson’s risk score

For Wilson’s risk score, this study analyzed 25 studies involving 12,601 patients, of which there were 1222 cases of difficult tracheal intubation. The pooled results showed a SEN of 0.42 (0.40–0.45), SPC of 0.81 (0.80–0.81), PLR of 4.18 (2.82–6.18), NLR of 0.56 (0.43–0.73), DOR of 7.93(4.37–14.4) and AUC of 0.7799, with a Q* index of 0.7185. See Fig. 7.

**Fig. 8:**
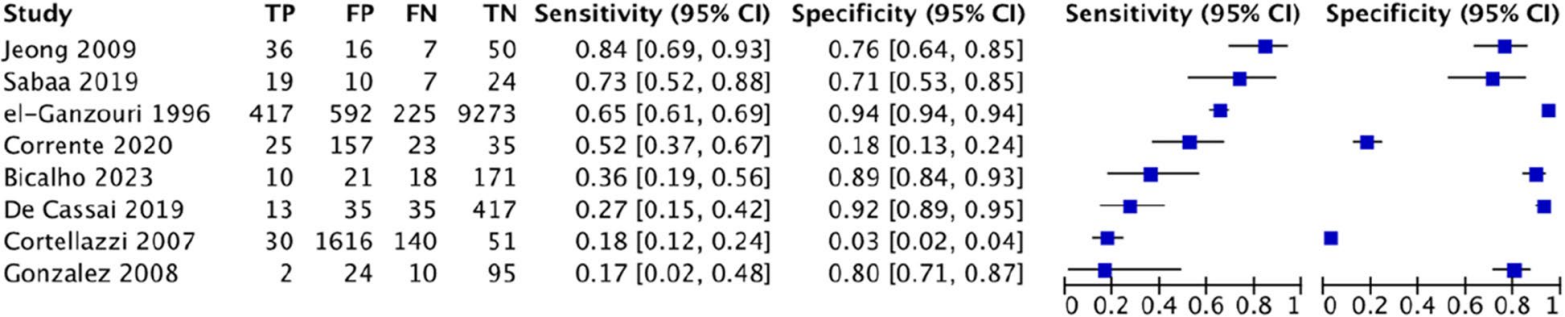
Forest plot of El-Ganzouri risk index

For El-Ganzouri risk index, this study analyzed 8 studies involving 13,604 patients, of which there were 1017 cases of difficult tracheal intubation. The pooled results showed a SEN of 0.54 (0.51–0.57), SPC of 0.8 (0.80–0.81), PLR of 1.79 (0.33–9.81), NLR of 1.05 (0.39–2.78), DOR of 1.72(0.09–31.77) and AUC of 0.4888, with a Q* index of 0.4916. See Fig. 8.

### Imaging test

**Fig. 9:**
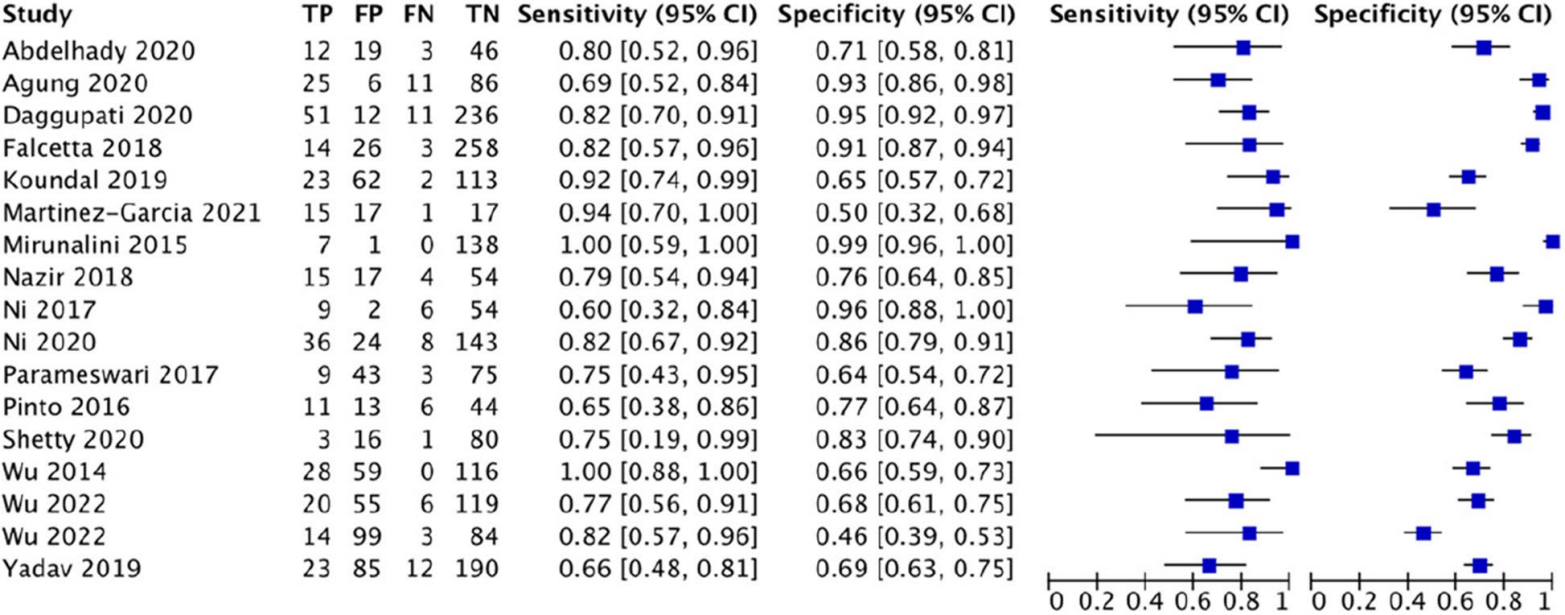
Forest plot of US-DSE

For US-DSE, this study analyzed 17 studies involving 2804 patients, of which there were 395 cases of difficult tracheal intubation. The pooled results showed a SEN of 0.80 (0.75–0.84), SPC of 0.77 (0.74–0.79), PLR of 3.97 (2.88–5.47), NLR of 0.3 (0.23–0.38), DOR of 17.25(9.55–31.17) and AUC of 0.8715, with a Q* index of 0.802. See Fig. 9.

**Fig. 10:**
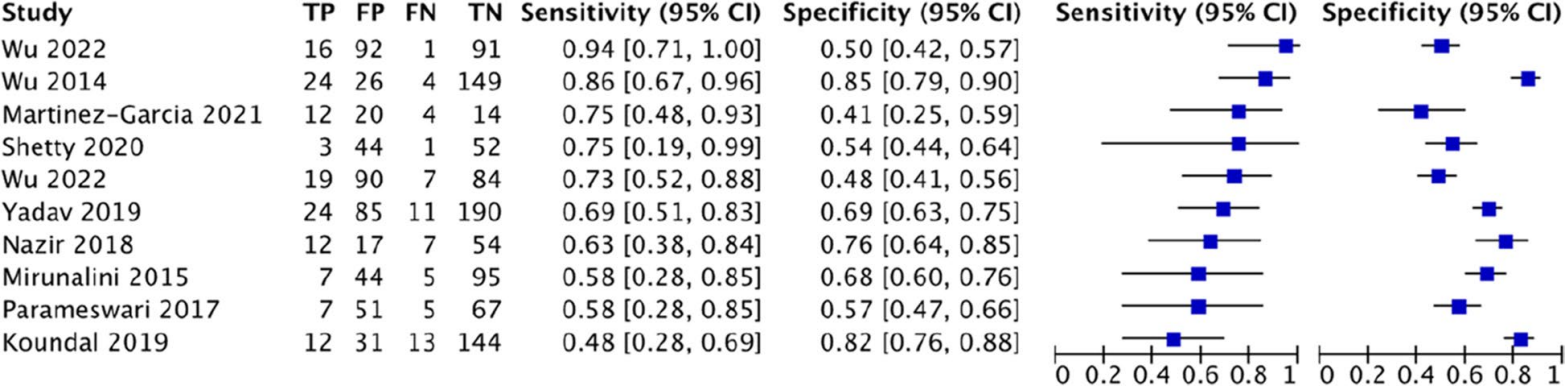
Forest plot of US-DSHB

For US-DSHB, this study analyzed 10 studies involving 1634 patients, of which there were 194 cases of difficult tracheal intubation. The pooled results showed a SEN of 0.70 (0.63–0.76), SPC of 0.65 (0.63–0.68), PLR of 2.04 (1.56–2.68), NLR of 0.51 (0.39–0.66), DOR of 4.61(2.69–7.89) and AUC of 0.7366, with a Q* index of 0.6824. See Fig. 10.

**Fig. 11:**
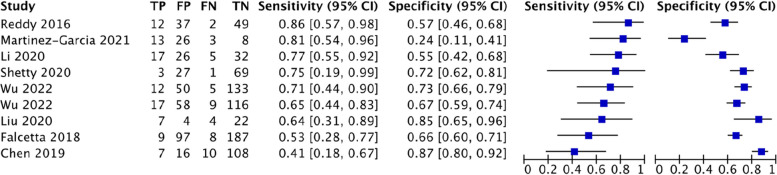
Forest plot of US-DSVC

For US-DSVC, this study analyzed 9 studies involving 1209 patients, of which there were 144 cases of difficult tracheal intubation. The pooled results showed a SEN of 0.67 (0.59–0.75), SPC of 0.68 (0.65–0.71), PLR of 1.96 (1.53–2.52), NLR of 0.56 (0.45–0.70), DOR of 4.06(2.72–6.06) and AUC of 0.7183, with a Q* index of 0.6676. See Fig. 11.

**Fig. 12:**
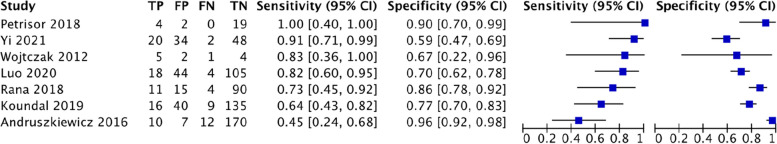
Forest plot of US-HMDR

For US-HMDR, this study analyzed 7 studies involving 831 patients, of which there were 116 cases of difficult tracheal intubation. The pooled results showed a SEN of 0.72 (0.63–0.80), SPC of 0.80 (0.77–0.83), PLR of 3.62 (2.48–5.28), NLR of 0.38 (0.26–0.56), DOR of 11.61(7.09–19.02) and AUC of 0.8378, with a Q* index of 0.7698. See Fig. 12.

**Fig. 13:**
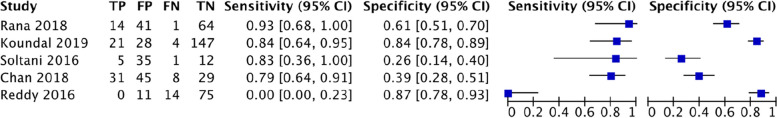
Forest plot of US-Pre-E/E-VC

For US-Pre-E/E-VC, this study analyzed 5 studies involving 586 patients, of which there were 99 cases of difficult tracheal intubation. The pooled results showed a SEN of 0.72 (0.63–0.80), SPC of 0.80 (0.77–0.83), PLR of 3.62 (2.48–5.28), NLR of 0.38 (0.26–0.56), DOR of 11.61(7.09–19.02) and AUC of 0.8378, with a Q* index of 0.7698. See Fig. 13.

The detailed results of each prediction method can be seen in Table 2.

**Table 2:**
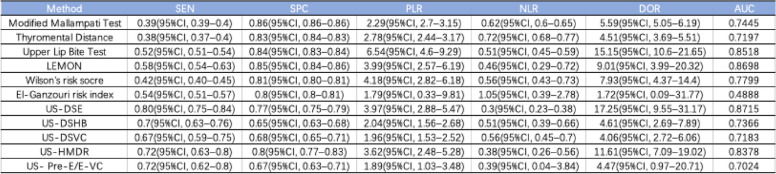
Prediction Methods Accuracy Results

### Reporting Biases

According to the Spearman correlation coefficient and the shape of the SROC curve, it can be concluded that there is no significant threshold effect in the accuracy evaluation of difficult airway intubation prediction methods included in this meta-analysis. However, non-threshold effects are present in each prediction method included, and there is significant heterogeneity in the pooled SEN, SPC, PLR, NLR and DOR of each method. As a result, we used a random effects model for meta-analysis.

This study used meta-regression to identify the sources of significant heterogeneity resulting from non-threshold effects. Possible covariates such as patient demographics (age, height, weight, and BMI), study design(case control or not), blind(blinded or not), sample size(< 100 or ≥ 100) [12] and obese(obese population or not) were analyzed using bivariate models.

The Meta-regression results are showed in Supplementary fig S1. The sources of potential heterogeneity cannot be determined for most prediction methods. (*P* > 0.05). However, for MMT, sample size may be the primary cause of heterogeneity (*P* = 0.02); for TMD, studying obese populations specifically could be the main source of heterogeneity (*p* = 0.0315); for ULBT, being a case control trial might be the primary cause of heterogeneity (*p* = 0.0068); for LEMON, conducting a blinded study could be the main source of heterogeneity (*p* = 0.02); and for Wilson’s risk score, conducting a blinded study could be the main source of heterogeneity (*p* = 0.0139).

**Fig. 14:**
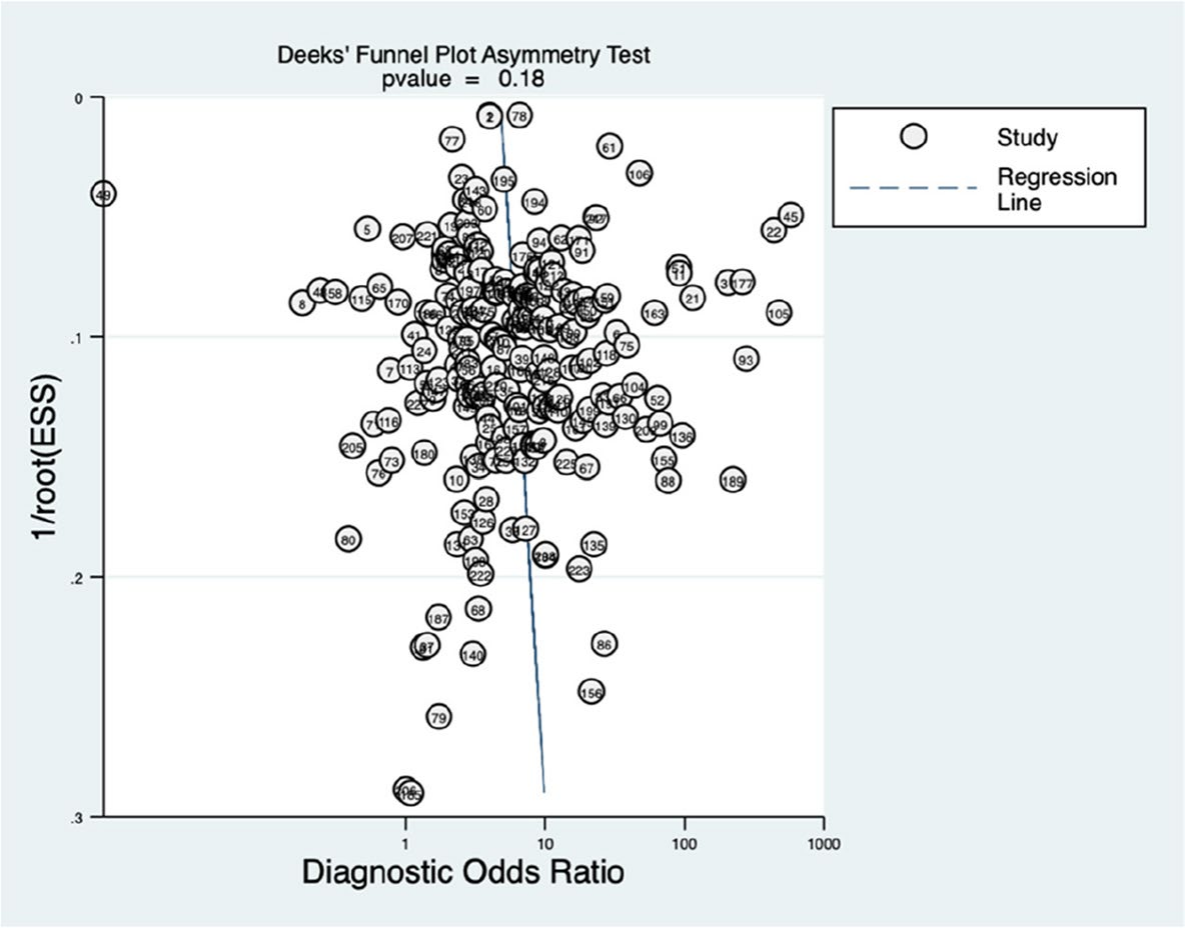
Deek's funnel plot of publication bias

This study used Deek's funnel plot asymmetry test to evaluate publication bias, and Fig. 14 displays the results which indicate no significant bias (*p* > 0.01).

## Discussion

The challenge of predicting difficult tracheal intubation has been a longstanding concern in the realm of anesthesiology. The consequences of an unanticipated difficult airway can be profound, ranging from prolonged surgical times to severe patient morbidity. This systematic review and meta-analysis was methodically conducted to synthesize the extant literature pertaining to diverse predictive methodologies, thereby furnishing a holistic evaluation of their diagnostic precision.

### Physical examination

Conventional methods, notably the Modified Mallampati Test, have long been entrenched in clinical paradigms due to their non-invasive nature and expedient application. However, the derived pooled sensitivity of 0.39 for this particular test underscores its potential limitations, particularly in its capacity to comprehensively identify patients predisposed to difficult intubation scenarios. As for the Thyromental Distance, it shares similar advantages to the Modified Mallampati Test, but its pooled sensitivity of 0.38 also renders it unsuitable as a standalone method for the assessment of difficult tracheal intubation The results for these two methods align with those derived from Roth's study [8]. As for the Upper Lip Bite Test, the pooled sensitivity obtained in this study was 0.52, significantly lower than previous similar studies [8]. Such discrepancies might arise due to variations in sample sizes or differences in the inclusion criteria for the literature. In summary, all three aforementioned physical examination methods exhibit high specificity and low sensitivity, making them unsuitable for sole reliance in predicting difficult tracheal intubation.

### Multivariate scoring system

Composite indices, such as the LEMON score, are designed to amalgamate multiple clinical variables, aiming for a comprehensive assessment. However, a pooled sensitivity of 0.58, while an improvement over some standalone physical examination methods, still presents challenges. Similar results are also reflected in Wilson’s risk score and the El-Ganzouri risk index, with this study's derived pooled sensitivities being 0.42 and 0.54, respectively. These data suggest that while multivariate scores provide a broader perspective, they are not foolproof and should be used in conjunction with other assessment tools.

### Imaging test

The incorporation of imaging techniques, with an emphasis on ultrasound-based methodologies, represents a paradigmatic shift in predictive strategies. The US-DSE method, boasting a pooled sensitivity of 0.80, underscores the promise inherent in these techniques. Their capacity to proffer granular anatomical insights in real-time is unparalleled. Recent studies, such as the meta-analysis by Carsetti et al., have further validated the use of ultrasound in airway assessment. Carsetti et al. found that ultrasound can be a reliable predictor of difficult direct laryngoscopy, supporting our findings on the effectiveness of ultrasound-based methods. Their study emphasizes the potential of incorporating advanced imaging techniques into routine preoperative assessments to enhance predictive accuracy [236]. However, it's imperative to acknowledge the operator-dependent nature of these modalities, which necessitates rigorous training to ensure consistent efficacy. Moreover, at the current stage, there is no standardized method for using ultrasound equipment to predict difficult tracheal intubation, nor a defined cut-off point. There is also insufficient data to prove the true effectiveness of such predictive methods. Therefore, the establishment of standardized testing procedures for these methods, the determination of cut-off points, and further in-depth research are essential.

### Future Directions

The nexus of medical technology and data analytics holds immense promise. The potential integration of artificial intelligence and deep learning algorithms, trained on expansive datasets, could revolutionize predictive accuracy. These algorithms could discern intricate patterns or correlations, potentially overlooked in traditional assessments. For example, Tavolara’s study proposed a deep learning model designed to identify patients who are difficult to intubate using frontal face images, leveraging an ensemble of convolutional neural networks. The proposed model outperforms traditional bedside tests, achieving an AUC of 0.7105 [237]. Hayasaka’s study utilized convolutional neural networks to link patients' facial images with intubation difficulty, creating an AI model capable of classifying intubation difficulty. This model achieved an accuracy of 80.5%, with an AUC of 0.864 [238]. Moreover, the exploration of patient-centric factors, such as genetic markers, proteomic profiles, or even biomechanical attributes, could further refine predictive models. Currently, there are studies targeting specific patients or diseases, using biomarkers to predict difficult tracheal intubation. For instance, Iacovazzo's study assessed the correlation between the likelihood of a difficult airway occurrence and the Insulin-like Growth factor 1 (IGF-1) levels in patients with GH-producing pituitary adenoma. The findings underscored a pronounced correlation between high IGF-1 levels and the occurrence of difficult airway [239].

### Limitations

This systematic review and meta-analysis encounter several limitations that must be acknowledged. A significant limitation is the heterogeneity of the included studies, with variations in patient demographics, study designs, and definitions of difficult tracheal intubation contributing to this heterogeneity. The lack of a standardized definition for difficult tracheal intubation across studies introduces potential bias and variability in the results. While most studies define Cormack-Lehane (CL) grades III and IV as indicators of difficult tracheal intubation, this definition only identifies difficulty in vocal cord visualization during direct laryngoscopy and does not necessarily equate to difficult tracheal intubation [8]. Relying solely on CL grading may introduce bias despite its high correlation with difficult tracheal intubation [7]. Some studies define difficulty based on the number of intubation attempts, but this approach is highly dependent on the clinician's skill level.

Additionally, the operator-dependent nature of certain techniques, such as ultrasound-based methods, necessitates rigorous training and standardization to ensure consistent efficacy. Differences in cutoff points among prediction methods and variations in clinician ability can further complicate the interpretation and comparison of findings. The potential for publication bias remains another limitation, despite the use of Deeks' funnel plot to assess it, as studies with negative or inconclusive results may be underreported.

Future research should address these limitations by standardizing definitions and methodologies, ensuring rigorous training for operator-dependent techniques, and exploring advanced technologies to improve predictive accuracy. By mitigating these limitations, future studies can provide more reliable and generalizable evidence for the prediction of difficult tracheal intubation.

## Conclusions

This systematic review and meta-analysis evaluated various preoperative prediction methods for difficult tracheal intubation in adult patients without obvious airway abnormalities. The findings indicate that no single method demonstrates unequivocal superiority in predictive accuracy. Traditional physical examination methods, such as the modified Mallampati test, thyromental distance, and upper lip bite test, exhibit high specificity but low sensitivity, limiting their utility as standalone predictive tools.

Multivariate scoring systems, including the LEMON score and Wilson’s risk score, provide a more comprehensive assessment by integrating multiple clinical variables, yet their sensitivity remains moderate. Imaging techniques, particularly ultrasound-based methods like the distance from the skin to the epiglottis, show higher sensitivity and specificity, suggesting their potential in enhancing predictive accuracy. However, the effectiveness of these methods is influenced by factors such as operator skill and the lack of standardized procedures and cut-off values.

The existing evidence underscores the need for a synergistic approach that combines various predictive techniques tailored to specific patient demographics and clinical contexts. Future research should focus on integrating advanced technologies, particularly artificial intelligence and deep learning algorithms, to improve predictive models. Additionally, exploring patient-specific factors, such as genetic markers and biomechanical attributes, could further refine these models.

For clinical practice, it is crucial to standardize testing procedures and establish clear cut-off values to enhance the reliability and accuracy of preoperative difficult airway prediction. Implementing a multi-modal predictive approach in clinical settings may reduce the incidence of unanticipated difficult intubations, thereby improving patient safety and outcomes.

In conclusion, a synergistic approach combining multiple predictive methods tailored to individual patient profiles offers the most promising direction for future research and clinical application. Standardizing procedures and leveraging technological advancements are essential steps towards better management of difficult airway predictions.

### Supplementary Information

Supplementary  Materials 1.
Supplementary  Materials 2.

## Data Availability

The datasets used and/or analysed during the current study are available from the corresponding author on reasonable request.
