# Supplementary material for: Evaluation of preoperative difficult airway prediction methods for adult patients without obvious airway abnormalities: a systematic review and meta-analysis
Source: BMC Anesthesiol. 2024 Jul 17;24:242. doi: 10.1186/s12871-024-02627-1 (PMC11253413; doi:10.1186/s12871-024-02627-1)

**Supplementary**

**Search strategy used for the searches (using PubMed search engine**): ((((((((((test[Title/Abstract]) OR (tests[Title/Abstract])) OR (exam[Title/Abstract])) OR (examination[Title/Abstract])) OR (predict[Title/Abstract])) OR (predictor[Title/Abstract])) OR (assessment[Title/Abstract])) OR (exam[Title/Abstract])) OR (physical examination [ Title / Abstract ])) or management [ Title / Abstract ]) AND ((((((difficult airway [ Title / Abstract ]) or difficult intubation [ Title / Abstract ]) or difficult face mask ventilation [ Title / Abstract ]) or difficult laryngoscopy [ Title / Abstract ]) Or difficult tracheal intubation [ title / abstract ])) or airway management [ title / abstract ]).

**China National Knowledge Infrastructure (CNKI) and Wanfang Database were searched electronically.**

The following terms will be included:

1. 气道管理
2. 困难气道
3. 困难气道评估
4. 超声
5. 预测方法
6. Difficult airway
7. Predictor
8. Difficult intubation
9. Ultrasound

Reference lists of eligible studies and review articles will be reviewed.

No limitations will be imposed on publication dates.

**Search strategy used for the searches (using Cochrane Library search engine**):

ID Search

#1 MeSH descriptor: [Airway Management] explode all trees

#2 MeSH descriptor: [Intubation] explode all trees

#3 Difficult airway

#4 Difficult tracheal intubation

#5 assessment

#6 evaluation

#7 predictor

#8 Mallampati

#9 LEMON

#10 Ultrasound

#11 upper lip bite test

#12 Wilson’s risk score

#13 El-Ganzouri risk index

#14 #3 or #4

#15 #5 or #6 or #7

#16 #8 or #9 or #10 or #11 or #12 or #13

#17 #14 and #15

#18 #14 and #15 and #16

**EMBASE database were searched for the following terms:**

"predictors", "prediction" and "risk factors" of "difficult intubation"and "difficult airway".

**Fig S1**: Quality assesment of the incuded studies using QUADAS-2


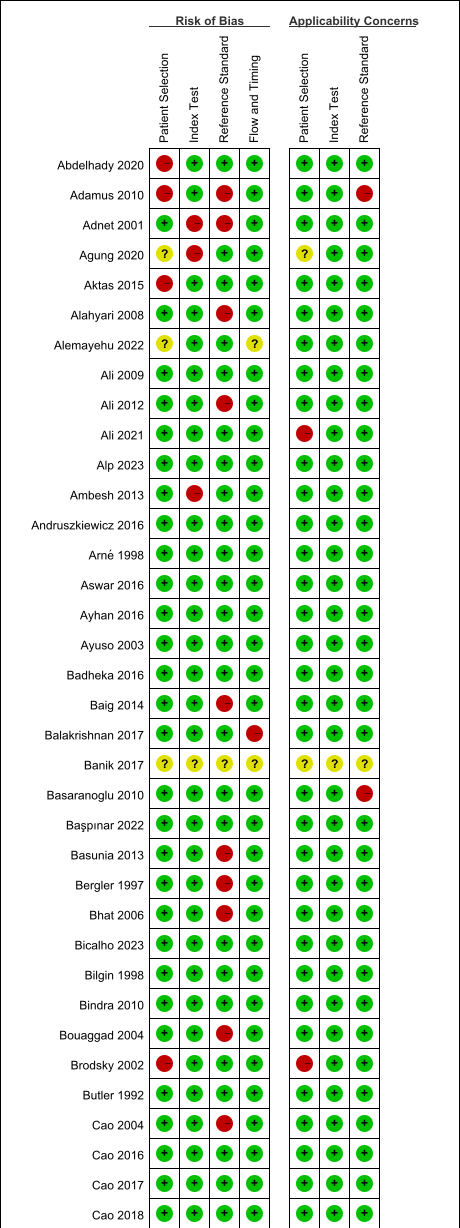


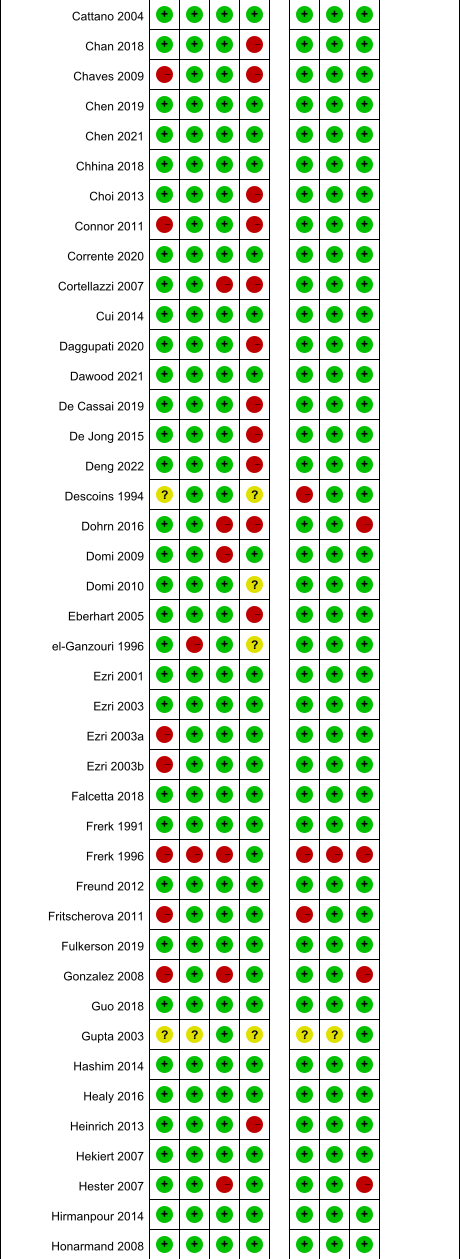


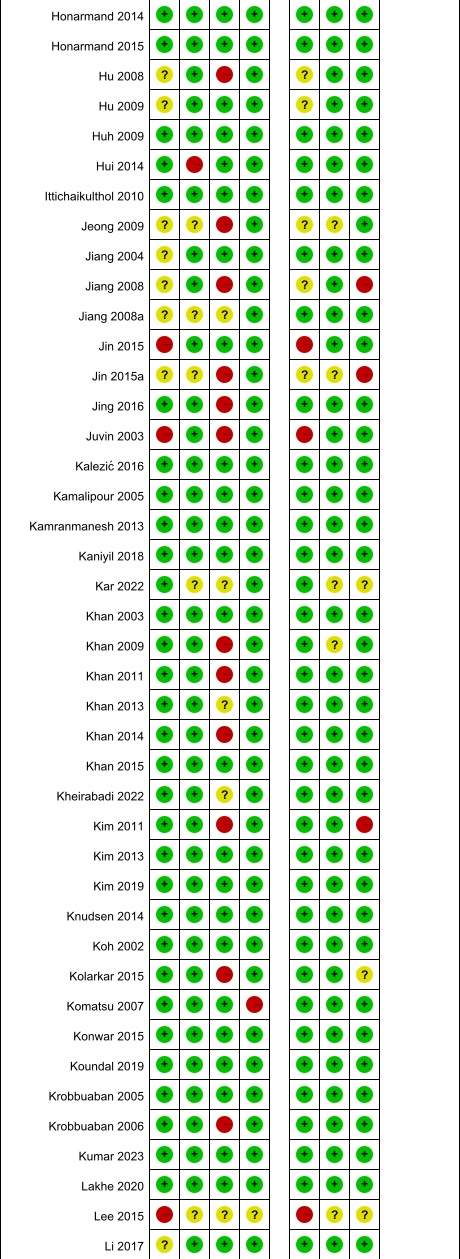


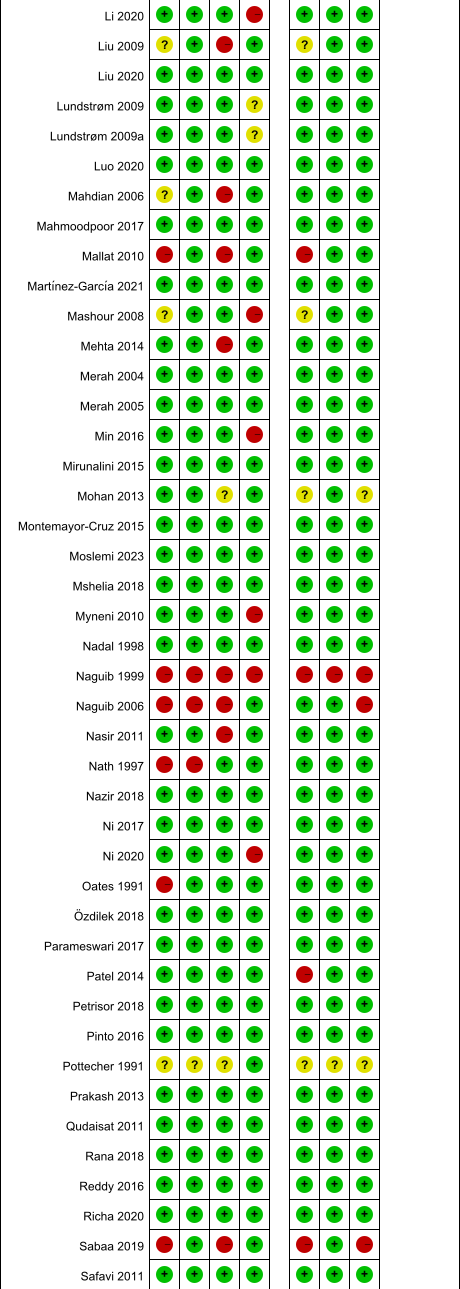


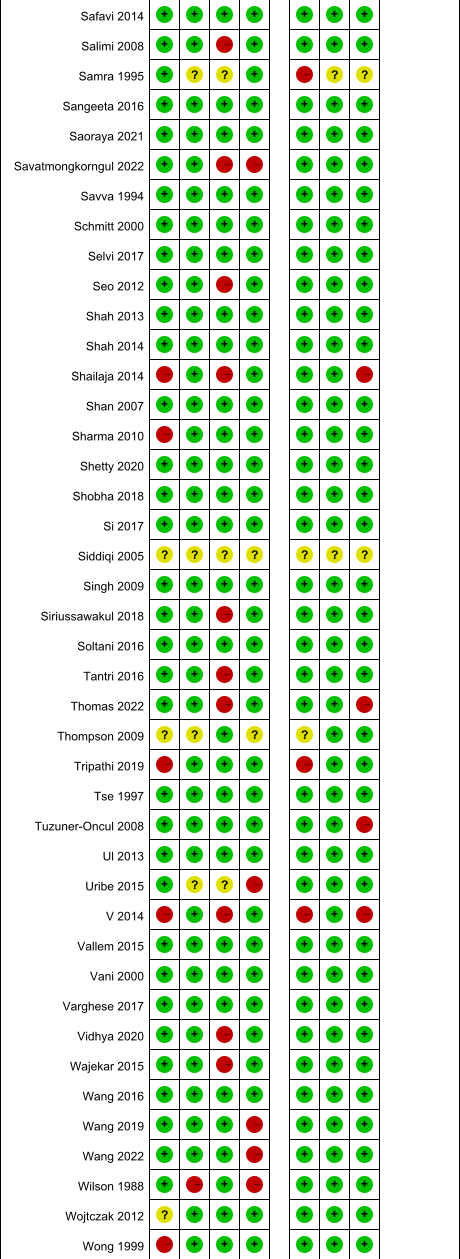


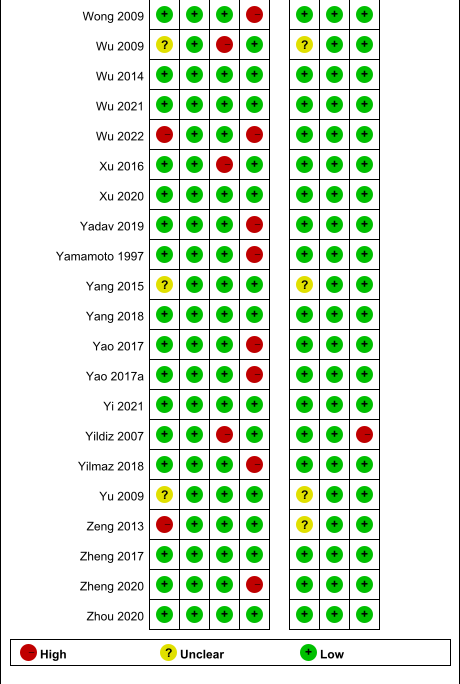


- **Fig S2:** Meta-regression results for each prediction methods


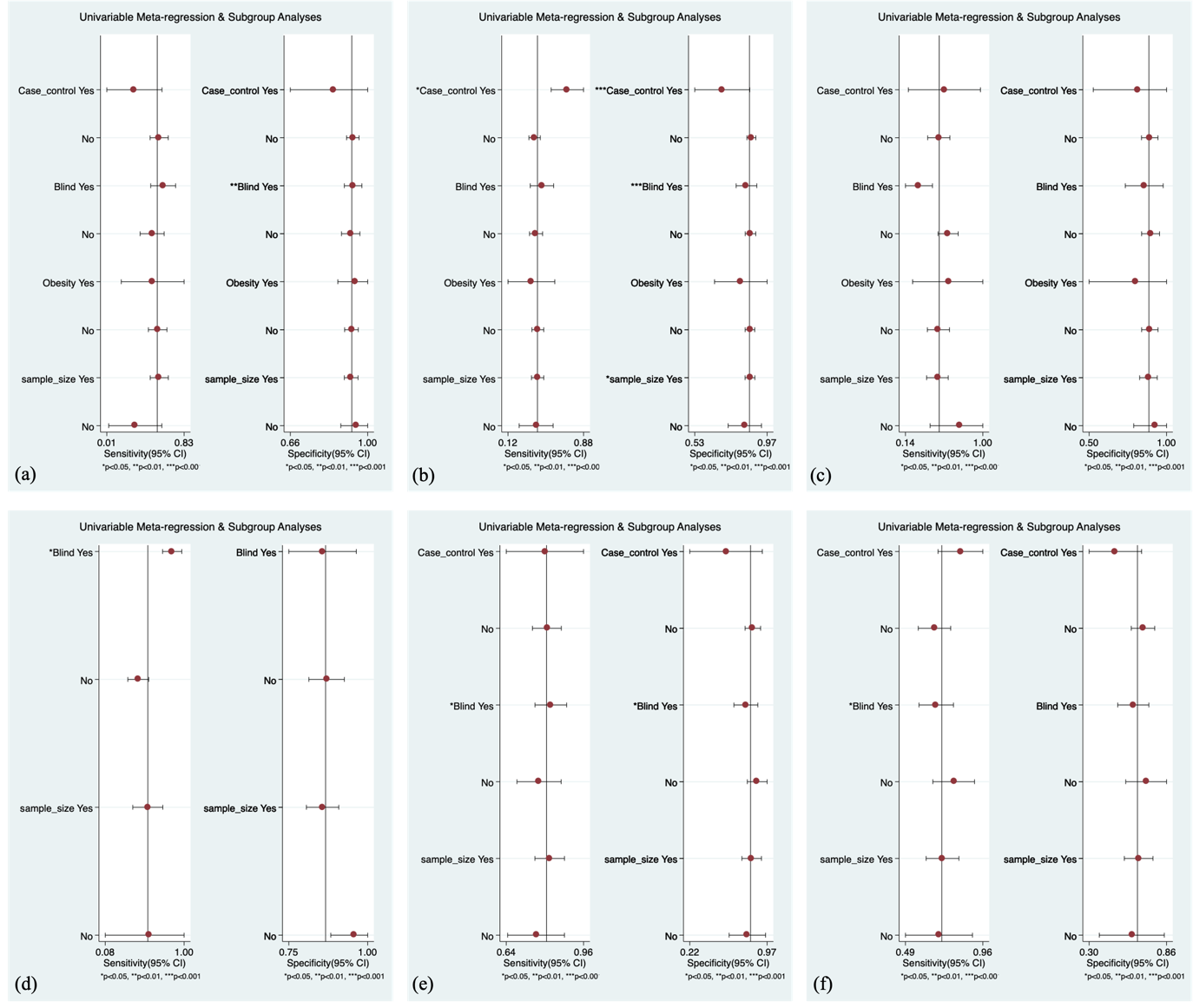

Supplement: Supplementary file 1 — Supplementary Materials 1. Supplementary Materials 2. [file 12871_2024_2627_MOESM1_ESM.docx]
